# Development and Evaluation of a Hybrid Course in Clinical Virology at a Faculty of Pharmacy in Lille, France

**DOI:** 10.2196/10766

**Published:** 2019-04-11

**Authors:** Anne Goffard, Pascal Odou, El Moukhtar Aliouat, Cécile-Marie Aliouat-Denis, Christophe Carnoy, Bertrand Décaudin, Cuny Damien, Mounira Hamoudi, Claire Pinçon, Katia Quelennec, Sebastien Zanetti, Pierre Ravaux, Annie Standaert

**Affiliations:** 1 Université de Lille, Centre National de la Recherche Scientifique, INSERM, Centre Hospitalier Universitaire Lille Institut Pasteur de Lille, U1019 - UMR 8204 – Centre d'Infection et d'Immunité de Lille Lille France; 2 EA 7365 - Groupe de Recherche sur les Formes Injectables et les Technologies Associées Lille France; 3 Université de Lille, Centre Hospitalier Universitaire Lille Institut Pasteur de Lille, EA 4483 - Impact de l'Environnement Chimique sur la Santé Humaine Lille France; 4 Université de Lille, INSERM, Centre Hospitalier Universitaire Lille U1008 - Controlled Drug Delivery Systems and Biomaterials Lille France; 5 Université de Lille, Centre Hospitalier Universitaire Lille EA 2694 - Santé Publique: Épidémiologie et Qualité des Soins Lille France; 6 Lille University Faculty of Pharmacy and Biology of Lille Lille France; 7 Université de Lille, INSERM, Centre Hospitalier Universitaire Lille UMR995 - Lille Inflammation Research International Center Lille France

**Keywords:** virology, pharmacy, education, distance, e-learning

## Abstract

**Background:**

During their studies, pharmacy students must acquire the specific skills in clinical virology required for their subsequent professional practice. Recent experiments on teaching and learning in higher education have shown that hybrid courses strengthen the students’ commitment to learning and enable high-quality knowledge acquisition.

**Objective:**

This study concerned the design and deployment of a hybrid course that combines face-to-face and Web-based instruction in clinical virology for fourth-year pharmacy students. The study’s objectives were to (1) measure the students’ level of involvement in the course, (2) gauge their interest in this type of learning, and (3) highlight any associated difficulties.

**Methods:**

The study included 194 fourth-year pharmacy students from the Lille Faculty of Pharmacy (University of Lille, Lille, France) between January and June 2017. The students followed a hybrid course comprising an online learning module and 5 tutorial sessions in which professional situations were simulated. The learning module and 3 online evaluation sessions were delivered via the Moodle learning management system. Each tutorial session ended with an evaluation. The number of Moodle log-ins, the number of views of learning resources, and the evaluation marks were recorded. The coefficient for the correlation between the marks in the online evaluation and those in the tutorials was calculated. The students’ opinions and level of satisfaction were evaluated via a course questionnaire.

**Results:**

The course’s learning resources and Web pages were viewed 21,446 and 3413 times, respectively. Of the 194 students, 188 (96.9%) passed the course (ie, marks of at least 10 out of 20). There was a satisfactory correlation between the marks obtained in the online evaluations and those obtained after the tutorials. The course met the students’ expectations in 53.2% of cases, and 57.4% of the students stated that they were able to work at their own pace. Finally, 26.6% of the students stated that they had difficulty organizing their work around this hybrid course.

**Conclusions:**

Our results showed that pharmacy students were strongly in favor of a hybrid course. The levels of attendance and participation were high. However, teachers must be aware that some students will encounter organizational difficulties.

## Introduction

### Background

A degree in pharmacy can lead on to various professions such as community pharmacists, hospital pharmacists, medical biologists, pharmacists in industry (production, quality control, marketing, etc), and regulatory pharmacists. Pharmacy students choose their career track progressively, as they complete internships and are exposed to learning experiences in the course of their studies. In France, these vocational studies are organized around an initial 3-year diploma in pharmaceutical science and then a 2-year specialist diploma [1]. Pharmacy students then choose between 2 options for the third and final part of their initial training: a short cycle for future community pharmacists and those working in industry or a long cycle for future hospital pharmacists and medical biologists. In our faculty of pharmacy, third-year students receive lectures and tutorials in general virology. During the fourth year, the clinical virology teaching is delivered in the hybrid format described in this study. At the end of the fourth year, students commit to a career track for 1 to 5 years. Pharmacy studies are, therefore, lengthy; during this time, the teaching staff monitor the acquisition of specific professional skills while encouraging the students to get involved in the learning process throughout the course.

#### Information and Communication Technology in Education and Electronic Learning

Recent developments in the field of information and communication technologies (ICT) for education and the now widespread availability of ICT tools have opened up new possibilities for initializing and continuing education in the health care professions. Historically, education in this field was based on a teacher lecturing to relatively passive students in a lecture room. Over the last few years, student-focused teaching has been developed by using interactive systems (eg, voting buttons in the lecture room), serious gaming, professional simulations, and hybrid courses that combine online and in-class learning [2]. A large number of studies performed in various health care sectors have shown that hybrid courses (1) facilitate the acquisition of in-depth knowledge via online lectures and evaluations and (2) enable the development of professional skills during face-to-face teaching (the latter can be organized around debates, group presentations, practicals, simulated professional exercises, and commented readings of texts) [3-6]. Hybrid courses appear to reinforce the students’ commitment to learning and enable the high-quality acquisition of knowledge [7]. A recent meta-analysis compared blended learning with more conventional courses; blended learning was found to be associated with greater acquisition of skills, particularly in health care professionals—a group whose commitment to gaining new skills is rarely doubted [8]. Among undergraduates, the level of motivation for acquiring new skills and experiences has a key role in academic success [9]. Although hybrid courses appear to increase the levels of satisfaction and motivation among nursing and medical students, we are not aware of studies conducted among undergraduate pharmacy students [10,11].

#### Hybrid Courses in Virology

Medical virology lends itself well to hybrid courses in which complex theoretical knowledge can be acquired online and know-how can be developed during face-to-face teaching sessions. Innovative teaching and learning techniques in virology are now emerging; they range from picture-card and memory-card methods [12] to the reverse classroom [13] and the creation of virtual viruses [14].

### Study Objectives

This study focused on a hybrid course in clinical virology for fourth-year pharmacy students at the Lille Faculty of Pharmacy (University of Lille, Lille, France). The study’s objectives were to (1) quantify the students’ involvement in a hybrid course, (2) assess the students’ level of interest in this type of learning, and (3) highlight any difficulties encountered during this new type of course. A convergent study design (as defined by Creswell and Plano Clark) was used to assess relationships between quantitative variables (such as connection time, course marks, etc) and qualitative variables [15].

## Methods

### Study Population

**Figure 1:**
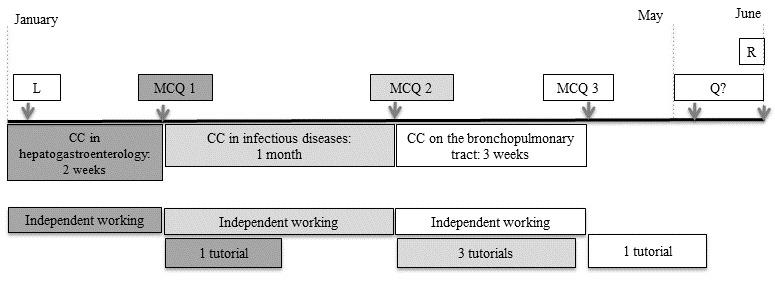
Schematic diagram of the hybrid course. Coordinated courses (CCs) in hepatogastroenterology are indicated by dark gray boxes; CCs in infectious disease indicated by light gray boxes; and CCs on the bronchopulmonary tract are indicated by white boxes. L: opening lecture; MCQ: online multiple choice questionnaire; Q?: period during which the students could evaluate the course via a questionnaire; R: retake exam.

The hybrid virology course was developed for fourth-year pharmacy students. The study took place during the year’s second semester, that is, between January and May (Figure 1). A total of 210 fourth-year students were registered for 2017.

### Description of the Hybrid Course

Fourth-year pharmacy studies are organized as coordinated courses (CCs) in several disciplines. The CCs take place between January and May; a 2-week CC in hepatogastroenterology is followed by a 4-week CC in infectious diseases and then a 3-week CC on the bronchopulmonary tract. For the sake of consistency, the course’s independent (online) working and in-class tutorials were organized in the same manner (Figure 1).

The course module was made available on the Moodle learning management system (LMS), which was hosted on the Lille Faculty of Pharmacy’s server. It began with a conventional lecture during which the lecturer presented the teaching and earning objectives, the course’s timeline (notably the dates of the in-class teaching), the evaluation procedures (based on the continuous appraisal of coursework), the Moodle LMS (through which the students could access the learning resources), the examination sessions, and a discussion forum. All the registered fourth-year pharmacy students were entered by default for the course module on the Moodle LMS, which they could access by using their university username and password. At the same time, the students could order paper copies of all the learning resources via the system at the end of the presentation lecture and could then collect the paper copies 4 days later.

### Online Learning Resources

The learning resources were made available to all the registered students via the dedicated zone on Moodle. The course was based on textbooks in clinical virology [16-18] by using the Opale (scenari.org) multimedia management tool and a template document to simultaneously generate a paper copy, a website that could be uploaded to the Moodle platform, and sets of multiple-choice questions (MCQs) [19]. The course covered all the knowledge to be acquired via short videos, downloadable summary documents produced by the lecturer, and links toward free accessible articles on the internet (ie, enriched content). The course’s structure mirrored that of the CCs (Textbox 1). For each CC, the learning objectives were clearly specified. For each virus in the course, the knowledge was distributed into sections covering some general background on virology, the basics of epidemiology, the clinical presentations, the diagnostic tools, and the principles of patient management (prevention and treatment). For each viral syndrome, the virus responsible, the specific clinical and diagnostic features, and the principles of treatment and prevention were detailed. The learning resource was presented to the students as a photocopy on which tags for enriched content on Moodle and on the internet were indicated. Along with the learning resource prepared by the lecturer, additional documents were available for consultation (vaccination schedules, articles from specialist journals, summary sheets, etc).

### Online Evaluations

At the end of an independent learning period (corresponding to a CC), knowledge acquisition was assessed via an online set of 20 MCQs that had to be completed in under 20 min. A total of 3 MCQs were administered between January and May (Figure 1). After connecting to Moodle, the student had up to 20 min to complete the MCQs; after that time, the results were recorded and the session closed automatically, regardless of whether or not the student had answered all the questions. For each student, 20 MCQs were randomly drawn from a database containing 40 to 70 questions, depending on the course. The MCQ session could be accessed over a 5-day period.

### In-Class Tutorials

At the end of an independent learning period, students were invited to attend a total of 5 in-class tutorials (these sessions lasted 1.5 hours and consisted of 25 to 30 students at a time). During the first part of the session, the lecturer answered the students’ questions. In the second part, the students were invited to participate in role-playing games that simulated practical situations encountered by community pharmacists. The learning objective was for the students to be able to apply their knowledge to simulated professional practice and to best assist patients during drug dispensing and/or the provision of advice on preventing viral infections (treatment goals, procedures for administering antiviral drugs, advice on vaccination, etc). For each practical situation, the students formed groups of 3 and took turns playing the roles of the pharmacist, the patient, and the observer. The student playing the role of the patient was given a written description of the practical situation and the associated expectations. The student playing the role of the observer was given an evaluation grid for noting specific aspects, such as the pharmacist’s general attitude, the answers to the patient’s questions, and the specific information on drug dispensing and/or prevention that the patient should receive in the scenario. At the end of the scenario, the observer reported his/her observations to the other players. A total of 2 different scenarios were simulated in each session, and the students changed roles for each situation. After the 2 scenarios had been completed, the students debriefed with the lecturer. At the end of each tutorial session, each student had to fill out a written evaluation (lasting about 10 min) in the form of 2 questions on the topic with short, open answers.

**Learning resources within the hybrid module (section, subsection, and chapters).**

Coordinated course on gastroenterohepatology

- Viruses
  - Hepatitis A virus
  - Hepatitis B virus
  - Hepatitis C virus
  - Hepatitis E virus
  - Gastroenteritis virus
- Syndromes
  - Viral gastroenteritis

Coordinated course on infectious diseases

- Viruses
  - Herpes virus
  - Enterovirus
  - Human immunodeficiency virus
  - Papillomavirus
  - Rubella
  - Measles
  - Mumps
- Syndromes
  - Viral meningoencephalitis

Coordinated course on the bronchopulmonary tract

- Viruses
  - Influenza
  - Syncytial respiratory virus
- Syndromes
  - Bronchiolitis in the newborn
  - Upper respiratory tract infections
  - Lower respiratory tract infections

### Evaluation of Coursework

The learning module was evaluated by averaging the 3 scores from the online MCQ marks and the 5 evaluations completed at the end of the tutorials (the tutorial marks). An average score of 10 or more out of 20 constituted a pass. Students who failed the continuous appraisal were asked to attend a retake examination in June; this took the form of an online 20-min, 20-MCQ session in an examination hall and then a 1-hour written examination with 2 questions.

#### Data Collection and Analysis

The study took place between January and June 2017. Several types of data were collected:

1. To assess the students’ participation in the online learning module, we recorded the time connected to the course’s Web page in each session, the number of forum messages, the total number of learning resource views per month, and the number of students having consulted each teaching resource between January 13, 2017, (the date on which the module was opened), and June 30, 2017, (the date on which the module was closed).
2. To assess the students’ level of success in the course module, the marks obtained in the MCQ sessions and in the evaluations performed at the end of each tutorial session were recorded, and the mean value was calculated for each student.
3. To quantify the students’ opinion of the hybrid course, we analyzed the answers to a modified version of the lecture evaluation questionnaire that has been used at the University of Lille for the last few years. The study questionnaire comprised all the usual questions plus an additional question on any difficulties that the students had encountered. There were 18 closed questions and 2 open questions. The students completed the questionnaire online (on the Moodle platform) between April 4, 2017, and May 30, 2017.

### Statistical Analyses

The coefficient *r* for the linear correlation between the MCQ marks and the tutorial marks was calculated, and the residuals were analyzed using IBM SPSS Statistics for Windows software (version 19.0, IBM Corp, Armonk, NY) [20]. After a linear model (Y=aX+b) had been built, the residuals were calculated as the difference between the marks obtained and those predicted by the model. Residual values above 3 or below −3 were considered to be aberrant. This analysis enabled us to detect and then exclude 8 aberrant values.

The time spent connected to the course’s Web page was analyzed by calculating Pearson coefficient *r* for the linear correlation with the overall mark on one hand and the in-class *mark* on the other hand. The coefficient *r* ranges between −1 and 1: the further away the value is from 0, the more significant the correlation. Depending on the value of *r*, a correlation *t* test was also performed.

### Ethical Approval

The students were informed that their Moodle data and questionnaire answers would be collected and analyzed for research purposes. The collection of all data on the University of Lille’s LMS has been registered with the French National Data Protection Commission (Commission Nationale Informatique et Libertés, Paris, France).

## Results

### Characteristics of the Study Population

A total of 210 (114 females and 96 males) fourth-year pharmacy students were registered for 2017. Furthermore, 16 of the 210 registered students were repeating their fourth year and had already completed a virology course a year earlier. Hence, the study population comprised 194 students.

### Consultation of the Learning Resources

The Moodle log-ins and forum messages were tracked. A total of 8 messages were posted on the forum by the lecturer and 5 were posted by the ICT technician. None of the students posted a message on the forum.

Between January 13, 2017, and June 30, 2017, 194 students viewed the course’s Web page for a median (range) of 9.213 (0-98.847) seconds. The learning resources were viewed 21,446 times by the 194 students, that is, an average of 110.5 log-ins per student. The highest number of views per month (n=8849) was observed in January. This number decreased progressively until April (the month when the in-class tutorial sessions finished), with 1991 views. The views in May (n=287) and June (n=134) concerned the students convened for the retake examination.

The course’s Web page was viewed 3413 times by the students during the course period. Furthermore, 174 of the 194 students viewed the additional learning resources at least once. The most frequently viewed documents concerned viral hepatitis (the French national guidelines on the management of hepatitis C infections, a treatment summary, and a vaccination schedule). The least frequently viewed documents concerned the measles-mumps-rubella. All the students ordered a set of photocopies online and collected it.

### Course Pass Rates

The mean mark obtained for the 3 online MCQ sessions was 13.9 out of 20. The mean mark obtained for the evaluations performed at the end of the tutorial sessions was 14.4 out of 20 (range 1.2-19). Moreover, 11 of the 194 students participated in less than 4 of the 5 tutorial sessions. Overall, the mean mark for the continuous appraisal (MCQs and post-tutorial evaluations) was 14.0 out of 20 (range 0.6-18.6). The online MCQ sessions enabled us to assess the knowledge acquired by the students during the independent work periods, and the post-tutorial evaluations provided a guide to the professional skills acquired during the simulation exercises. The linear correlation coefficient *r* for the 2 types of marks was 0.466 (n*=* 193; *P*<.001). In view of the large sample size and the external factors that could influence these marks (the level of attendance, the marks obtained in the subject in the previous year, the commitment to personal work and to revision, etc), we considered that the correlation between the 2 types of marks was satisfactory. After eliminating the aberrant values via a residual analysis, the correlation coefficient *r* was .483, emphasizing the satisfactory correlation between the 2 means. Our results revealed a correlation between the level of the theoretical knowledge and the quality of the professional skills acquired.

Furthermore, 15 students scored below 10 out of 20, and so were asked to attend the retake examination. Moreover, 5 students did not attend, and 1 of the 10 attendees failed the examination. Hence, 188 of the 194 students (96.9%) passed the course module. Finally, 188 of the 194 students completed the questionnaire on the hybrid course, corresponding to a response rate of 96.9%.

With a view to evidencing a correlation between the time spent consulting the online version of the course and the final examination mark, we extracted the time spent connected to the course’s Web page; the correlation was not statistically significant. In fact, the linear correlation coefficient was *r*=0.15 (*P*=.07) when the full set of observations was taken into account. However, the number of the connection times were clearly too long (more than 3 hours) or too short (a few minutes) and therefore could not be considered as objective measures of the amount of work. We, therefore, limited our correlation analysis to *reasonable* connection times of between 30 min and 90 min, but the correlation was again not statistically significant (*r*=−0.173; *P*=.40). The same result was obtained when considering the *in class mark* with the whole dataset (*r*=0.123; *P*=.15) and with *reasonable* page connection times alone (*r*=−0.070; *P*=.66).

### The Students’ Opinion of the Course

**Figure 2:**
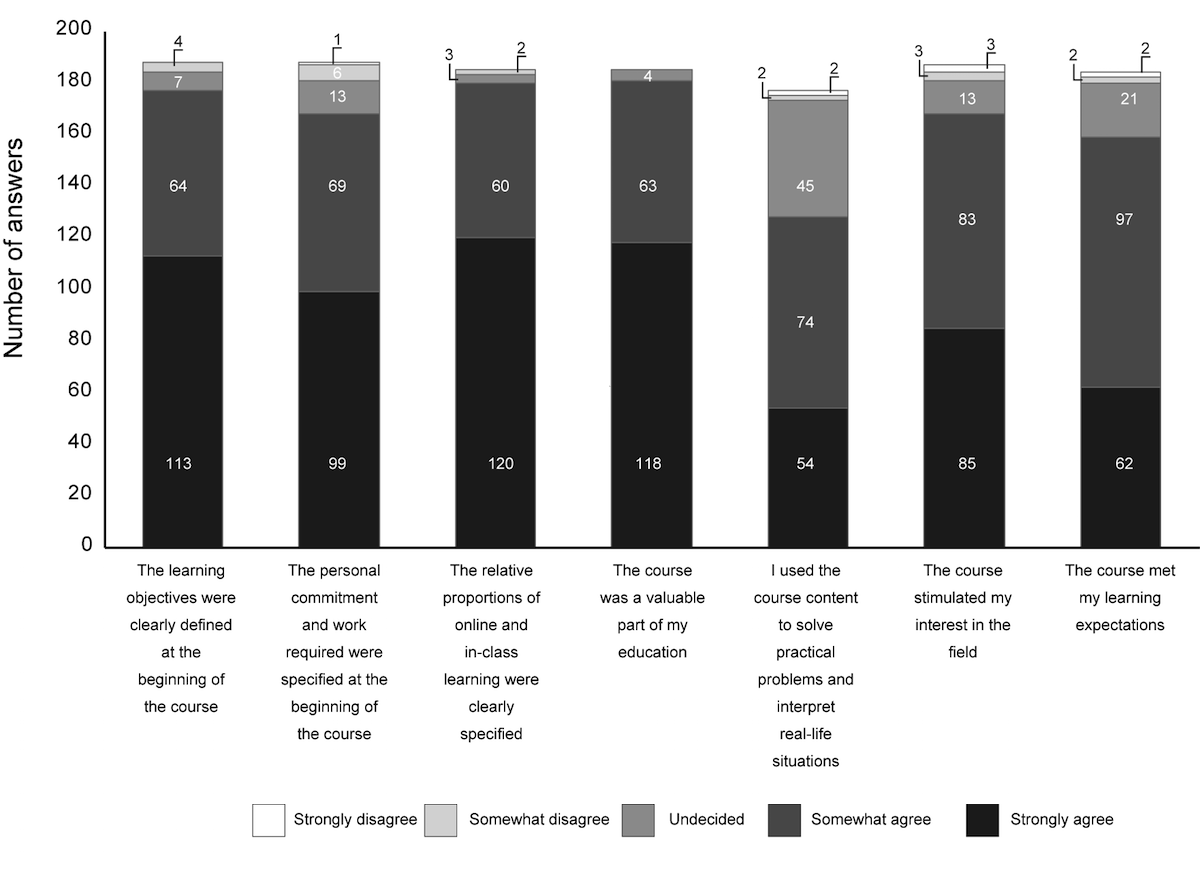
The students’ opinions of organizational aspects of the hybrid course, and the hybrid course’s impact on the value of the teaching in general and the virology teaching in particular.

A total of 4 questions addressed the module’s structure (Figure 2). Furthermore, 60% of the students considered that they had been well informed about the course’s learning objectives and that the proportion of independent learning required had been accurately specified. The students generally considered that the relative proportions of online work and in-class tutorials had been explained and that the evaluation procedures had been well described. A total of 21% of the students stated that they had not consulted any of the learning resources on Moodle, and 26% of the students stated that they had consulted all the resources.

In addition to the online teaching, 5 in-class tutorial sessions had been based around simulations of professional situations, so that the students could apply the theoretical knowledge acquired online to practical situations in a context managed by the lecturer. Moreover, 2 questions dealt with the hybrid course’s impact on the students’ level of interest in their studies in general and the discipline covered (clinical virology) in particular (Figure 2). The students considered that this type of course was a valuable part of their education and had increased their level of interest in clinical virology. The great majority considered that the course had met their expectations in terms of learning.

**Figure 3:**
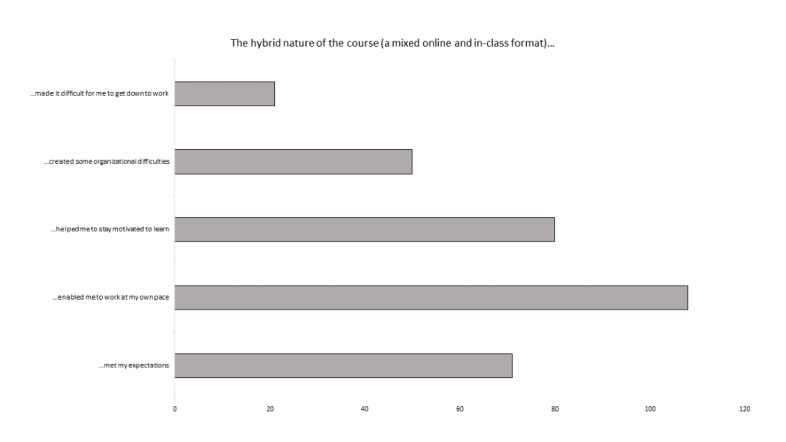
The students’ evaluation of the hybrid course, with regard to the level of motivation and work structure. The students could tick several items in the list.

Finally, a smaller majority (64%) considered that the hybrid course had helped them to solve practical problems. The hybrid course obliged the students to work more independently. Furthermore, 1 of the questionnaire items focused on any difficulties that the students may have had in this respect (Figure 3). A student could tick more than 1 answer, if he/she so wished. Only 53% considered that the course had met their educational expectations. On the whole, the students considered that the course had enabled them to work at their own pace and that it had boosted their motivation to learn. However, difficulties in getting organized and getting down to work were mentioned.

## Discussion

### Main Conclusions

Faced with low levels of attendance at lectures and with a view to offering new learning methods, we developed and deployed a hybrid course in virology for the first time in our faculty. We noted a high level of commitment from the students, with over 21,000 log-ins on the learning platform and high levels of attendance at the tutorial sessions. In fact, only 11 students attended less than 4 of the 5 tutorial sessions, although they passed the online MCQ sessions. At the end of the course, 15 students failed the module by failing to obtain a mark of 10 or more out of 20. These 15 included the 11 students who had not attended the majority of the tutorial sessions. However, after the retake examination, only 6 of the students failed the course overall. There was no correlation between the time spent viewing the online course and the final examination result. However, 98% of the students registered for the course received a paper copy. Hence, the page connection time did not necessarily reflect the time spent studying the course material. These results suggest that the paper copy alone enabled the students to acquire the knowledge evaluated in the examination.

In the feedback questionnaire, the students considered that this type of teaching met their expectations. Even though the hybrid course constituted the first experience of hybrid teaching for the majority of participants, the level of participation was high and enabled the students to pass the course evaluations.

During the course, the students were confronted with simulated professional scenarios for the first time. Our results showed a satisfactory correlation between the theoretical knowledge acquired during independent working and the students’ ability to acquire professional skills. These data are in line with the many studies showing that professional simulations improve the level of preparation for working life [21,22]. The degree of correlation between the theoretical knowledge and the acquisition of the professional skills might be further improved by building a predictive model of success in the course evaluations, which would notably take into account the students’ level of attendance at lectures, their average mark in virology in the previous year, and their average mark for the previous year as a whole. This type of predictive model might help to improve the students’ learning experience. In fact, certain students reported difficulties in work organization and independent working. To stimulate the students’ commitment and interest, these exercises should simulate a more diverse range of professional scenarios, reflecting the panel of skills required of pharmacists. During the pharmacy degree, more emphasis should be placed on professional simulations so that students understand the value of this exercise for their future profession.

The students consulted the learning resources (notably the short videos) very frequently (over 3000 views). This type of learning resource adds value for the students who view them. Our finding should encourage university teachers to produce this type of resource—even though this may require significant effort, along with assistance from the ICT staff. As also observed in the literature, we found that none of the students used the forum to interact with other students or with the lecturer [7]. The additional course materials were not extensively consulted, and their educational value should probably have been better explained. Finally, we confirmed Ladage et al’s [23] report that students like to receive a photocopy of the learning resources. The students then readily consulted the online learning resources to find more information on a tricky point or to gain an overview of the topic. This observation prompts us to think that when designing a hybrid course, the lecturer must offer different types of educational media [6].

Although hybrid courses are widely used in continuing professional education, they are not yet widely applied in undergraduate courses [24-26]. Students faced with novel learning methods must adapt and organize their working habits to meet the constraints of hybrid courses [7]. Even though our students considered that this type of course met their educational expectations and participated willingly, some reported (via the course questionnaire) difficulties in work organization and/or getting down to work. Given that the development of this type of course is strongly encouraged by universities, students should be provided with support (eg, through tutorials or courses on work methods,) throughout their degree course. The students liked the teacher-led tutorials, which appeared to help them plan their learning and to increase their level of enthusiasm for independent working [27,28]. A learning performance dashboard (indicating a student’s educational progression and the marks achieved in assessments, etc) might constitute a useful aid [29] as long as the teachers help the students to interpret the dashboard data [30]. In-class tutorial sessions enable discussion between students and the lecturers and among the students themselves, and also serve as an opportunity to acquire feedback on the acquisition of theoretical knowledge and the online exercises [16,17]. These in-class tutorials must be designed to promote interaction between the students and the lecturer and among the students themselves [18]. Finally, the in-class tutorials serve to reassure the students with regard to their learning strategies and to encourage them to pursue their efforts.

Lecturers developing hybrid courses could also benefit from support from electronic learning experts for both technical aspects (digital engineering) and educational aspects (learning engineering).

### Limitations

The implementation of the hybrid course was accompanied by the complete reorganization of our clinical virology courses for fourth-year pharmacy students: the replacement of lectures by online learning, an increase in the number of tutorial sessions (from 3 to 5), and the introduction of continuous appraisal. Hence, it was not possible to measure the learning impact of a hybrid course on the students’ academic success by comparing the results obtained by the students who attended the hybrid course with those of students who attended a conventional course the year before.

The hybrid module was used during a single academic year with a single-year group of students. The collection of data over several years would enable one to compare the level of performance from the 1-year group with the next and, potentially, to evaluate any changes in learning strategies in response to the above-mentioned organizational difficulties.

Our assessment of the correlation between the theoretical knowledge acquired during the independent working sessions and the professional skills acquired during the simulations highlighted a number of factors that might influence the students’ learning. Further analyses of these factors and the data collected in the hybrid module might enable us to build a predictive model of academic success and the acquisition of professional skills by the students in our faculty.

Furthermore, we did not find a significant correlation between the page connection time and the marks in the evaluations. In future research, we intend to transform the linear Web page into a nonlinear medium by adding tests that modulate the students’ progression through the course module. These tests (located at learning milestones) will enable students to evaluate their knowledge and will personalize each student’s progression through the module [31-33].

With regard to the online evaluations performed, the marks may have been biased by the students’ consultation of documents or by information swapping between students. However, the application of course assessments after the tutorial sessions may have countered this bias.

Our results showed that fourth-year pharmacy students were strongly in favor of a hybrid course and that the course met their educational expectations. This type of course enabled students to work at their rhythm, although teachers must be aware that some students will encounter difficulties organizing their work.

## Abbreviations

CC: coordinated course
ICT: information and communication technologies
LMS: learning management system
MCQ: multiple-choice question
